# P-1154. Prevalence and Antibiotic Resistance Patterns of Healthcare-Associated Infections at MCM Comprehensive Specialized Hospital in Ethiopia

**DOI:** 10.1093/ofid/ofaf695.1347

**Published:** 2026-01-11

**Authors:** Meti T Negassa, Erko Beyene, Lia Mogus, Wondmagegn Demsis, Jiksa Dhabessa

**Affiliations:** Johns Hopkins Bloomberg School of Pubic Health, Rockville, MD; University of Pisa, Pisa, Toscana, Italy; George Washington University, Virginia, Virginia; Myungsung Medical College, Addis Ababa, Adis Abeba, Ethiopia; Myungsung Medical College, Addis Ababa, Adis Abeba, Ethiopia

## Abstract

**Background:**

Healthcare-associated infections (HCAIs) significantly contribute to patient morbidity and mortality globally. Understanding causative agents and antimicrobial resistance is crucial for effective management but remains understudied in low-income settings, including Ethiopia. This study assesses the prevalence, antimicrobial resistance, and associated factors of HCAIs at MCM Comprehensive Specialized Hospital, Ethiopia.

**Methods:**

A retrospective chart review of 414 patients admitted from January 1 to December 31, 2022, was conducted using a structured electronic questionnaire. Data were analyzed using SPSS V.20.0 and summarized by frequencies and percentages. Binary logistic regression identified factors associated with healthcare-associated infections, with significance determined by adjusted odds ratios (AORs) and 95% confidence intervals (CIs).

**Results:**

The prevalence of healthcare-associated infections (HCAIs) was 13% (54/414), with the most common infections being urinary tract infections (21/54) and hospital-acquired pneumonia (17/54). Among the 54 HCAI cases, bacterial pathogens were detected and cultured in 34 patients. The most frequently identified bacteria were Coagulase-Negative Staphylococcus (9/34), Escherichia coli (8/34), and Enterococcus species (7/34). Antibiotic susceptibility testing revealed complete resistance to Ceftriaxone in 25/34 isolates and resistance to Cotrimoxazole in 33/34 isolates. Length of hospital stay exceeding 7 days, use of a urinary catheter, history of chronic respiratory disease, and previous antibiotic use were identified as significant factors associated with HCAI development.

**Conclusion:**

HCAI prevalence was high at MCMCSH, with common pathogens showing significant antimicrobial resistance. Findings highlight the need for early infection detection, improved prevention protocols, shorter hospital stays, antibiogram-guided antibiotic use, and targeted catheterization practices.

**Disclosures:**

All Authors: No reported disclosures

